# Smaller Size of Nesting Loggerhead Sea Turtles in Northwest Florida

**DOI:** 10.3390/ani16010071

**Published:** 2025-12-26

**Authors:** Matthew Ware, Luna Oliveira de Mello Vieira, Laura Fuentes-Tejada, Ian Silver-Gorges, Mariana M. P. B. Fuentes

**Affiliations:** Department of Earth, Ocean, and Atmospheric Science, Florida State University, Tallahassee, FL 32306, USA; luna.vieira@my.jcu.edu.au (L.O.d.M.V.); lfuentte154@doct.ub.edu (L.F.-T.); ian.silvergorges@ucf.edu (I.S.-G.); mfuentes@fsu.edu (M.M.P.B.F.)

**Keywords:** body size, *Caretta caretta*, curved carapace length, hatchling production, marine turtles, minimum size at reproductive maturity, nest site selection, threat assessment

## Abstract

The distribution of individual body sizes within a population can have substantial impacts on recovery estimates for endangered species. Recent studies have observed a reduction in the size of nesting sea turtles with potential implications for fecundity. To investigate the size of reproductive individuals and subsequent impacts on hatchling production for loggerhead turtles in northwest Florida, we evaluated a seven-year dataset from St. George Island, Florida, USA—the dominant nesting site in this region. Morphometric measurements and GPS locations were collected during nighttime surveys, whereas nest disturbances and hatchling production were reported from morning surveys. Mean minimum curved carapace length (CCL_min_) was 94.3 cm ± 5.7 cm SD (range: 80.2–109.1 cm). Out of 232 individuals, 9.1% fell below the 87 cm CCL_min_ threshold currently adopted by U.S. agencies as the minimum size at maturity. For each 1 cm increase in CCL_min_, an increase by 1 egg was observed in clutch size, and wash-out rates increased by 0.52%. Though the largest turtles laid the largest clutches, these nests also had a greater probability of wave wash-out, potentially moderating overall hatchling production from these individuals. These results highlight size-mediated factors related to fecundity—important elements for demographic modeling and management decisions.

## Simple Summary

Tracking body size distribution within a population provides useful information on demography, population growth trajectory, and size class- or stage-specific threats. Using multi-decadal datasets, several sea turtle monitoring programs have reported a global declining trend in the average body size of adult female turtles. This is concerning as smaller sizes could be an indicator of reduced foraging habitat suitability, which could have implications for reproductive output. In numerically small populations, such changes in reproductive output have the potential to slow population growth rates. This project investigates the current body size distribution of loggerhead sea turtles nesting in northwest Florida—among the smallest populations (numerically) in the southeastern United States—as well as body size-related consequences for nest site selection and hatchling production. Since 2016, approximately 9% of encountered turtles were below the minimum size at reproductive maturity currently adopted by U.S. management agencies. In addition, larger turtles laid larger clutches but also lost more nests to wave wash-out. Understanding the cause of reduced adult body sizes in sea turtles and its potential consequences for population-scale hatchling production is crucial for informed conservation management of these imperiled species.

## 1. Introduction

Body size varies significantly both among and within populations as an interaction between life history, habitat selection, foraging ecology, density dependence, environmental stochasticity, and individual variability [1,2,3,4,5,6,7]. Knowledge of the distribution of body sizes within a given population is crucial for accurate estimations of demography, evaluating trends in population growth, and quantifying size class- or stage-specific threats [8,9,10,11]. In particular, the minimum size at sexual maturity—the size at which individuals transition from nonreproductive juveniles to reproductively-capable adults—is a critical demarcation which influences reproductive outputs and impacts policy decisions for conservation actions [9,12,13,14,15,16]. For example, populations containing individuals who can reach sexual maturity sooner may have an increased lifetime reproductive output due to a longer reproductive stage duration. However, if the trade-off for this early maturation is a smaller body size, these individuals may experience lower fitness due to size-based competition for productive foraging sites and mates, greater risk of predation, and reduced fecundity per breeding event [17,18,19,20,21]. From a management perspective, if the transition into the “adult” stage is incorrectly defined, smaller reproductively-capable individuals may be at increased risk from threats because they may be misidentified in the field and misrepresented in population models, resulting in underestimates of both adult mortality and population-level reproductive output and overestimates of juvenile mortality rates [12,20,22,23,24,25,26].

In some species, life history characteristics may hinder our ability to collect such demographic data. For example, collection of morphological data from sea turtles (family Cheloniidae) is logistically challenging as they spend most of their life in the marine environment [12,27,28,29]. Therefore, most sea turtle population data is collected at nesting beaches when adult females come ashore to deposit eggs (e.g., [18,30,31,32,33,34,35]). Measurements of female carapace lengths collected on nesting beaches are then assumed to represent the range of possible body sizes for the “adult” life stage, an assumption extended to include unobserved adult males and non-reproductive adult females [36,37].

Size at maturity varies considerably amongst sea turtle species and populations and may change over time. For example, eastern Pacific and Mediterranean populations of leatherback (*Dermochelys coriacea*; Vandelli, 1761), green (*Chelonia mydas*; Linnaeus, 1758), and loggerhead turtles (*Caretta caretta*; Linnaeus, 1758) mature at smaller sizes than their western Pacific and Atlantic counterparts [34,38,39]. Gradients may be seen within a single basin as well, such as for Kemp’s ridley turtles (*Lepidochelys kempii*; Garman, 1880) and loggerhead turtles occupying the U.S. Gulf versus Atlantic seaboards [16,22]. Thus the “adult” body size threshold from one population may not accurately reflect other populations throughout the species’ range.

Recent publications have reported decreasing trends in mature female sea turtle body sizes across species, ranging from −0.5 cm/decade among hawksbill turtles (*Eretmochelys imbricata*; Linnaeus, 1766) on Milman Island, Australia [30], to −2.2 cm/decade among loggerhead turtles in Sal, Cabo Verde [18]. Such declining trends may be concerning as reproductive output is strongly correlated with maternal body size across taxa [17,40,41,42,43,44]. Smaller female turtles may thus have reduced fecundity compared to larger individuals [32,41,45,46]. The distribution of female body size within a population is not currently accounted for in sea turtle demographic models [14,37,47]. Typically, in stage-based modeling a singular mean clutch size per nest per female is used without explicitly including the relationship between body size and reproductive output (e.g., clutch size). However, with the declining trends in female body size reported across species and locations, this could result in an overestimation of the number of eggs laid each year and may misrepresent important or stochastic trends in model projections, including life stage-based threat assessments and relevant mitigation responses [48,49,50].

The interaction between body size, reproductive output, and habitat use or partitioning is another important consideration. Across taxa, larger individuals typically gain access to more productive foraging or breeding sites, which in turn improves offspring production or fitness [5,7,43,51]. Despite previous efforts relating female sea turtle body size to clutch and/or hatchling size (e.g., [45,52,53,54]) or individual preferences in nest microhabitat selection (e.g., [55,56,57,58]), there has been no exploration into how body size may affect fine-scale nest site selection. Poor intra-beach nest site selection by smaller, inexperienced, neophyte nesters or competitive exclusion of smaller turtles from higher quality sites could compound the physical limitations imposed by reduced body size on clutch size, resulting in further reductions in hatchling production or fitness.

Thus, an accurate definition of the minimum size at maturity and an understanding of the relationship between body size, habitat use, and hatchling production are crucial for informed population management [10,59,60]. For example, for loggerhead turtles in the northwest Atlantic Ocean, the U.S. National Marine Fisheries Service and U.S. Fish and Wildlife Service currently use a threshold size of 87 cm minimum curved carapace length (CCL_min_; the length from the nuchal scute to the notch between the supracaudal scutes measured along the spine using a flexible measuring tape) to differentiate between “subadult”—i.e., nonreproductive juveniles which have recruited to the neritic environment—and reproductively-capable “adult” turtles [47]. This value was based on length-frequency observations of nesting females throughout the southeastern U.S. primarily in the 1980s and early 2000s [8,37,54,61,62,63,64]. Since then, declining body size trends have been reported in the southeast U.S. [33], with observations as low as 74 cm CCL_min_ reported in the Northern Gulf of Mexico Loggerhead Recovery Unit [65]. This unit is among the numerically smallest genetically discrete populations of loggerhead turtles in the region [66], and faces significant threats at its nesting beaches such as high wave exposure, mammalian predation, coastal development, and artificial light [67,68,69,70,71]. This reduced population size and associated low nest density has made population-specific demographic parameters for this Recovery Unit time- and labor-intensive to collect. However, applying such parameters from other, larger populations to this Recovery Unit may hinder the accurate assessment of population trends, threats, and associated conservation actions needed to conserve this susceptible population [65,72].

Given that demographic parameters and threat exposure may vary geographically and temporally, it is crucial to tailor management plans with updated Recovery Unit-specific data to ensure effective conservation [12,47,72]. In this study, data collected from St. George Island, Florida, USA (i.e., the largest nesting assemblage in the Northern Gulf of Mexico Loggerhead Recovery Unit) over seven years is used to (1) evaluate the current size distribution of nesting loggerhead females on the island to compare to the minimum size at sexual maturity adopted by U.S. federal wildlife management agencies and to distributions reported from other beaches in the southeast U.S., and (2) assess whether female loggerhead body size correlates with nest site selection and any associated implications for hatchling production.

## 2. Materials and Methods

### 2.1. Study Site

**Figure 1:**
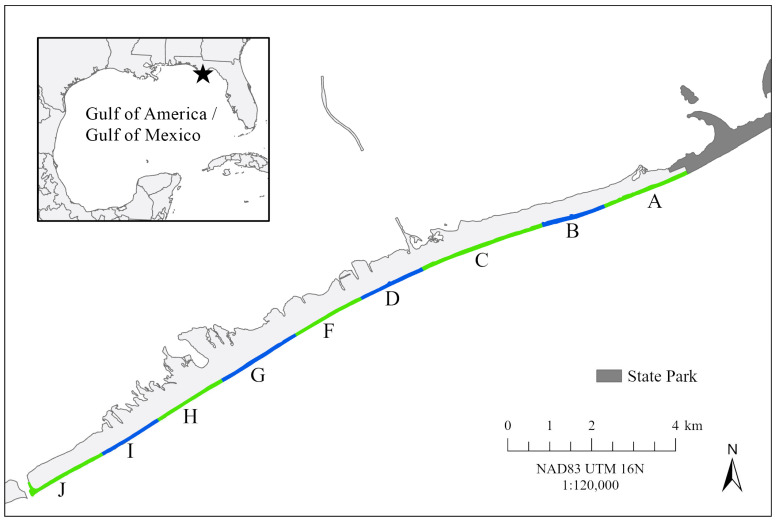
St. George Island, Florida, USA including the monitoring zones (A–J).

This study was conducted on St. George Island, Florida, USA (29.66° N, 84.86° W), a 33 km barrier island located in northwest Florida (Figure 1). The island is split into a 17.8 km populated region (western and central portion denoted by monitoring sections A–J in Figure 1) and the 15.2 km undeveloped Dr. Julian G. Bruce St. George Island State Park along its eastern portion. The populated portion of St. George Island hosts the most abundant nesting assemblage for the Northern Gulf of Mexico Loggerhead Recovery Unit with an average of 389.5 nests per year (±115.8 nests SD) [70,71,73]. Beaches on this part of the island are fairly uniform in profile with an average width of 58 m ± 5 m SD.

### 2.2. Beach Surveys

During the 2016–2024 nesting seasons (May–October, excluding 2020 and 2023), volunteers from the Apalachicola National Estuarine Research Reserve conducted morning nesting surveys within assigned sections of the beach (zones A–J in Figure 1). Each morning, they looked for new sea turtle crawls, demarcated new nests, and checked on existing nests following protocols established by the Florida Fish and Wildlife Conservation Commission [74]. If a nest was predated, washed over, washed out, or otherwise disturbed, volunteers noted the disturbance and provided commentary (e.g., how many eggs were lost, predator identity, name of the storm that caused the erosion). Predated nests were identified based on eggshells ejected onto the beach surface following consumption of the egg contents. Washed over nests were denoted by wet sand or the deposition of wrack material shoreward of the nest or active submersion of the nest was observed during morning patrols. A nest was considered washed out if it could not be found again after a high tide or storm event, and no subsequent hatchling emergence was observed from that nest location. Volunteers excavated nests either three days after emergence or at day 70 of incubation to calculate the clutch size (i.e., the number of eggs laid), hatching success (i.e., the number of hatched eggs divided by the number of eggs laid) and emergence success (i.e., the number of emerged hatchlings divided by the number of eggs laid) [74,75].

Female nesting rates peak on St. George Island starting in mid-June each year [70]. For one to two weeks during this time (Appendix A Table A1), morning surveys were supplemented by nighttime surveys conducted by the Florida State University Marine Turtle Research, Ecology, and Conservation Group (FSU MTRECG). From 21:00–04:00 local time, FSU MTRECG teams patrolled sections of the beach to intercept nesting females. For each turtle encountered, teams recorded the GPS location of turtle activities (e.g., nests, false crawls), checked for Inconel flipper and PIT tags, applied new tags as necessary, and recorded carapace measurements [76]. Carapace measurements collected with a flexible measuring tape included the minimum curved carapace length (CCL_min_, distance from the nuchal scute to the notch between the supracaudal scutes along the midline), “standard” or “notch-to-tip” curved carapace length (distance from the nuchal scute to the tip of the longest supracaudal scute along the midline), and curved carapace width (distance between the widest points of the carapace perpendicular to the midline) [77] Beach sections were not patrolled uniformly by FSU MTRECG personnel across all years to maximize encounter rates (Appendix A Table A1). Only loggerhead turtle nests corresponding to FSU MTRECG-intercepted females and subsequently monitored by Apalachicola National Estuarine Research Reserve volunteers were included in this analysis.

### 2.3. Statistical and GIS Analyses

Individual female body sizes across all years were initially pooled to visualize the overall data distribution and compare to the 87 cm CCL_min_ minimum size at maturity threshold adopted by U.S. federal wildlife managements agencies. Any interannual trends in mean female size and the proportion of females ≤ 87 cm CCL_min_ by year were evaluated using binomial (size class) or gaussian (CCL_min_) generalized linear models (GLMs). We excluded 23 repeated observations of the same individuals from the dataset and kept the smallest measurement for each turtle [31,78].

To describe general spatial patterns in the frequency of female emergences detected by size class across all years, rasterized kernel density estimates (KDEs) were generated with a search radius of 1300 m and a cell size of 25 m (each representing optimized values based on the observed dataset) using ≤87 cm and >87 cm size-class GPS observations separately in ArcGIS Pro v3.5.4 [79,80] (Esri; Redlands, CA, USA). The KDEs were then divided by the survey effort within each monitoring zone to generate spatial estimates per unit effort [81]. To account for differences in sample size between size classes, the resulting KDEs were then standardized to values between 0–1 using the equation:

(1) $$\frac{KDE\;cell\;value-minimum\;KDE\;value}{maximum\;KDE\;value-minimum\;KDE\;value}$$

The resulting KDEs thus represent relatively high- and low-density patterns in female emergence by size-class [81]. Differences in relative distribution were further visualized by subtracting the ≤87 cm size class standardized KDE from the >87 cm size class standardized KDE, such that more negative values suggest a greater relative use by the ≤87 cm size class and more positive values support greater >87 cm size class observations.

Spatial patterns in nesting female size were evaluated for statistical significance using generalized additive models (GAMs) including a spatial explanatory covariate in R v4.5.1 [82] (R Core Team; Vienna, Austria). GAMs were used to accommodate potential non-linear patterns associated with the spatial covariate in the form of a smoother. The GPS coordinates corresponding to females of a known size were transformed to the UTM 16N projected coordinate system to convert units from degrees to meters. The UTM coordinates were rotated clockwise 26.5°, the orientation of the long axis of St. George Island relative to the latitudinal parallel, to reduce the observed covariate dimensions from two (*x*- & *y*-axes) to one (*x*-axis only) [81]. Mean turtle size (continuous; CCL_min_) and size class (binary; ≤87 cm vs. >87 cm) were evaluated as a function of the beach location measured in kilometers from the rotated western UTM boundary in either a gaussian (CCL_min_) or binomial GAM (size class). Survey effort per monitoring zone was not included as a model offset in these GAMs as the models investigated the characteristics of encountered turtles, not their frequency of occurrence as in the KDE analyses, and the inclusion of effort resulted in reduced model performance and nonsensical units (e.g., mean CCL_min_ per unit effort).

The effect of female CCL_min_ or size class on hatchling production and threat exposure was explored through multiple metrics including clutch size, hatching success, emergence success, and rates of disturbance such as predation, invasion by sea oat (*Uniola paniculate*) roots, wave overwash, and wash-out. Depending on the form of the response variable, different GLM families were used including binomial (binary data such as predated vs. non-predated), negative binomial (count data such as clutch size), and beta (proportional data such as hatching and emergence success). Note that various disturbances are not mutually exclusive. For example, a clutch can be predated then washed over by a subsequent hurricane. A total of 29 nests (17.5%) were observed with multiple disturbances. When assessing rates of disturbance, such overlapping events were included to prevent undercounting disturbance rates. Few individuals (n = 10 out of 232 unique individuals) were observed laying multiple nests, so each nest was treated as a separate observation (i.e., maternal identity was not included as a random effect) as only one individual laid three nests, and the other nine individuals laid two nests each (representing a combined 13% of the available nesting data).

Additional assessments of nest distance from the mean higher high water elevation contour were conducted in ArcGIS Pro v3.5.4. Monitoring zone of a given nest was recorded during nighttime surveys; however, cross-shore nest distances from the recent high tide line or back-beach boundary were not. To extract cross-shore nest placement, nest GPS locations were measured to (1) the back-beach boundary (e.g., dune toe, upland construction) digitized from aerial imagery, and (2) the mean higher high water elevation contour derived from temporally averaged beach elevation profiles generated by repeated aerial LiDAR surveys [71,83]. Nest distance from the mean higher high water contour as a function of CCL_min_ was assessed using a gaussian linear model in R v4.5.1.

The statistical significance of explanatory variables was evaluated using likelihood ratio Chi squared, F-, T-, or Z-tests, as appropriate, comparing nested models with vs. without the variables of interest. A full description of the analyses, including model formulations and performance metrics, is available in the Supplementary Materials. Size class-based analyses are not included here in the hatchling productivity analyses given the low ≤ 87 cm sample sizes (Section 3.3, n = 12/166 nests, 7.2%); therefore, only analyses based on CCL_min_ are presented. Size class-based analyses are included in the Supplementary Materials with the understanding that results based on small sample sizes should be interpreted with caution.

## 3. Results

### 3.1. Proportion of Females < 87 cm CCL_min_

**Figure 2:**
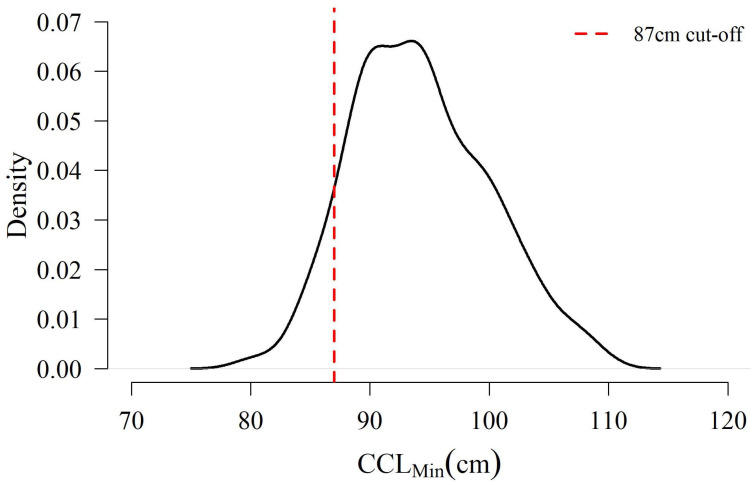
Distribution of 232 observed loggerhead minimum curved carapace lengths (CCL_min_) on St. George Island from 2016–2024 including the 87 cm delineation (in red) between subadult and adult size classes defined by National Marine Fisheries Service & U.S. Fish and Wildlife Service [47].

From 2016–2024, 232 unique turtles were available for analysis ranging in size from 80.2 cm to 109.1 cm CCL_min_ ($\bar{x}$ = 94.3 ± 5.7 cm SD, Figure 2). Of these individuals, 21 (9.1%) fell into the ≤87 cm size class. This proportion varied from 0% (n = 0/17 in 2024) to 16.3% (n = 7/50 in 2019), but year was not a significant predictor of either size-class assignment (χ^2^ *p* = 0.365) or mean female size (F test *p* = 0.717).

### 3.2. Spatial Emergence Patterns

**Figure 3:**
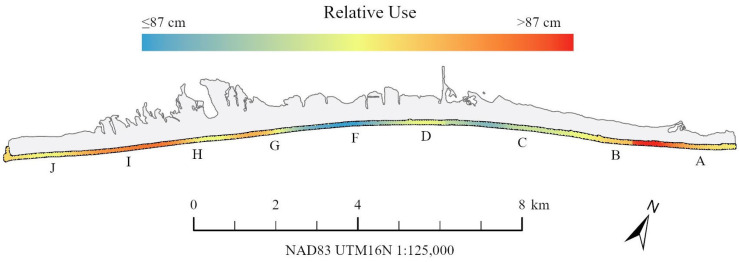
Relative use by ≤87 cm vs. >87 cm CCL_min_ size classes of loggerhead turtles on St. George Island, Florida, USA. Lettering beneath the island denotes the monitoring zone.

**Figure 4:**
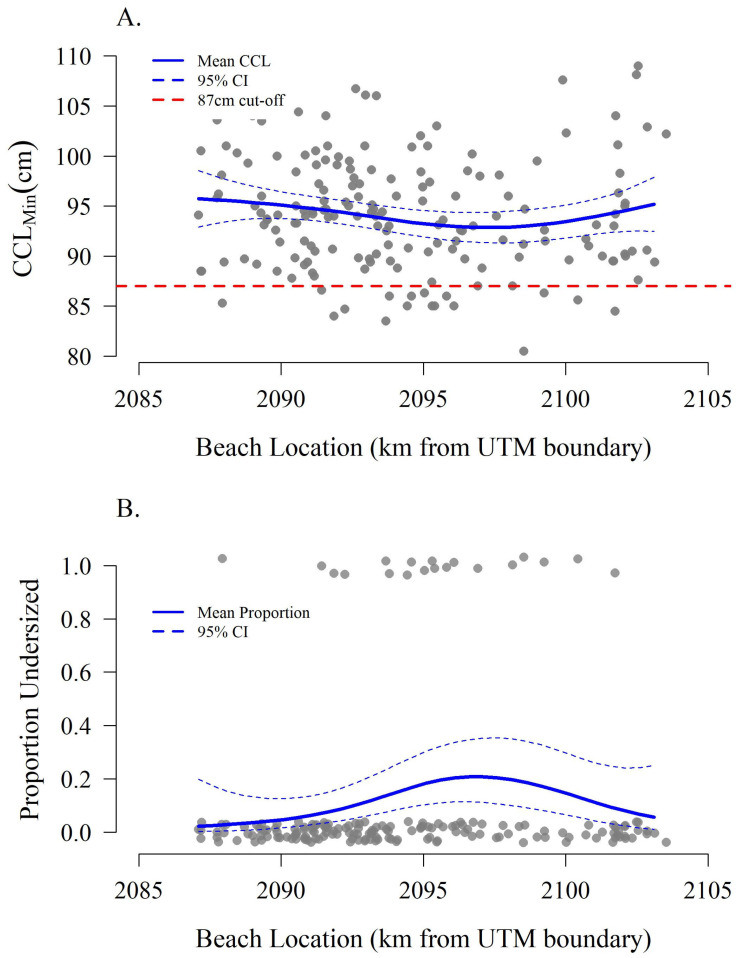
Mean CCL_min_ (**A**) and size class (**B**) by beach location measured as kilometers from the rotated western UTM boundary. Increasing distance indicates a movement from west to east along the island.

Of the 232 unique loggerhead turtles, 177 (76.3%) had GPS coordinates associated with their observations including 19 turtles ≤ 87 cm and 158 turtles > 87 cm. Standardized KDEs suggest potential differences in emergence location across the beach between size class with higher relative densities of ≤87 cm turtles emerging in the center-left portion of the island (zone F) versus the western (zones H–I) and eastern ends (zones A–B) for the >87 cm size class (Figure 3). Distances along the island corresponding to these zones show similar patterns in either mean CCL_min_ or proportion of ≤87 cm females (Figure 4). However, alongshore location was not statistically significant with respect to CCL_min_ (F test *p* = 0.170 by UTM distance, F test *p* = 0.375 by zone) or size class assignment by zone (χ^2^ *p* = 0.326 by Zone). Size class by UTM distance was probative (Figure 4B, χ^2^ *p* = 0.072).

### 3.3. Hatchling Production

**Figure 5:**
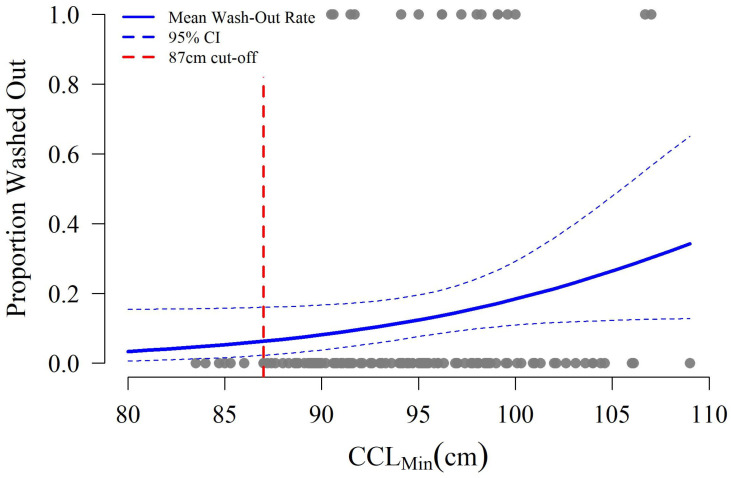
Wash-out rates as a function of female size (CCL_min_). The red dashed line denotes the current 87 cm threshold between subadult and adult size classes defined by National Marine Fisheries Service & U.S. Fish and Wildlife Service [47].

Most of the disturbances considered were not significantly related to turtle body size, including predation (Z test *p* = 0.951), root invasion (Z test *p* = 0.760), and wave overwash (Z test *p* = 0.374). The effect of CCL_min_ on exposure to disturbances was only suggestive with respect to wave wash-out rates (i.e., erosion, Z test *p* = 0.052), where modeled rates of wash-out increased by 0.52% per 1 cm increase in CCL_min_ (Figure 5). Based on this trend, the largest turtles (109 cm CCL_min_) could lose 34.5% of their nests to wash-out compared to 3.4% for the smallest individuals (80.2 cm CCL_min_). Wash-out rates were not linked to survey zone (all pairwise Z test *p* > 0.6), and larger turtles did not tend to nest closer to the mean higher high water elevation contour (T test *p* = 0.387).

**Figure 6:**
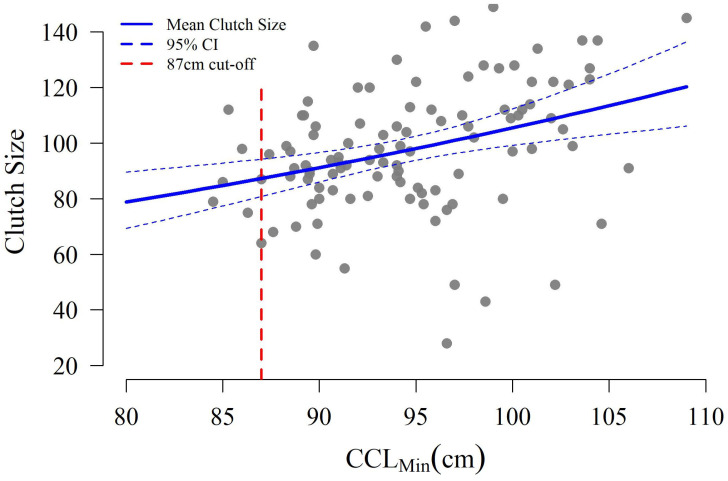
Clutch size as a function of female size (CCL_min_). The red dashed line denotes the current 87 cm threshold between subadult and adult size classes defined by National Marine Fisheries Service & U.S. Fish and Wildlife Service [47].

With respect to mean clutch size, nests which were predated (n = 39) or washed out (n = 16) were excluded as the initial clutch size pre-disturbance was not known, leaving 111 nests available for analysis. CCL_min_ was significantly related to clutch size (Z test *p* < 0.001), with clutch sizes increasing by 1 egg per 1 cm increase in CCL_min_ (Figure 6).

**Figure 7:**
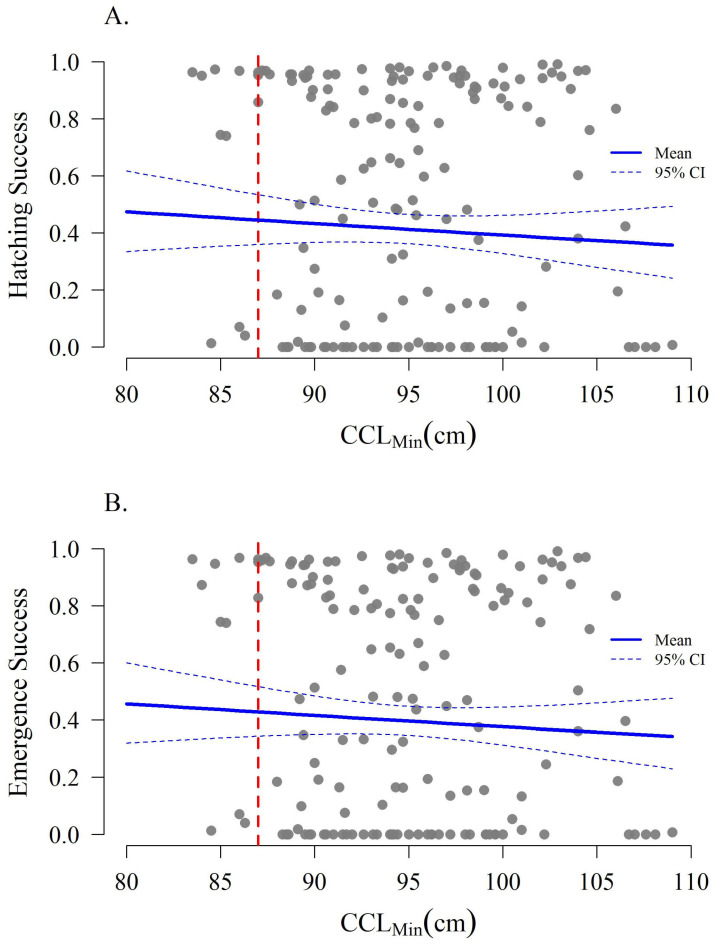
Hatching (**A**) and emergence success (**B**) from and all available nests as a function of female size (CCL_min_). The red dashed line denotes the current 87 cm threshold between subadult and adult size classes defined by National Marine Fisheries Service & U.S. Fish and Wildlife Service [47].

When considering completely undisturbed nests (n = 45), overall mean hatching success was 82.6% ± 22.6% SD and emergence success was 81.0% ± 22.4% SD. Minimum curved carapace length was not significantly related to either hatching (Z test *p* = 0.785) or emergence success (Z test *p* = 0.975). However, when disturbances were included, overall mean hatching and emergence success declined to 51.9% ± 40.4% SD and 49.9% ± 40.0% SD, respectively. Under these circumstances, hatching and emergence success trended −0.50% per 1 cm increase in body size but these trends were not statistically significant (Z test *p* = 0.369 and 0.371, respectively, Figure 7).

## 4. Discussion

Of the unique individuals identified nesting on St. George Island (n = 232), 9.1% were below the current threshold of 87 cm CCL_min_ used by U.S. federal wildlife management agencies to denote “adult” loggerhead turtles (Figure 2). This rate is consistent with another nesting site for this Recovery Unit in Gulf Shores, Alabama from 2011 to 2017, which observed 9.6% of nesting females to be ≤87 cm [65]. Taken together, this consistency suggests that the minimum size at reproductive maturity for loggerhead turtles in the region may need to be reconsidered [33,65,72]. Ensuring an accurate definition of the minimum size at sexual maturity is critical for population management as individual counts, threat assessments, mortality assignments, and fecundity estimates may be attributed to the wrong life stages, hindering viability projections [9,14,25]. This is particularly important for small or isolated populations, as their diminutive size or restricted connectivity increases their risk of local extinction [59,60].

The Northern Gulf of Mexico Loggerhead Recovery Unit is among the smallest in the Northwest Atlantic distinct population segment for loggerhead turtles and its range significantly overlaps several major threats including commercial trawling and oil and natural gas extraction [37,68]. To understand the population-level impacts of these activities and inform any necessary management action, the frequency and life stage of affected individuals must be accurately reflected in population model parameters such as survivorship. Previous modeling work has demonstrated that older juvenile and adult life stages hold a greater value for population recovery than eggs and hatchlings given their higher annual survival rates, but it is these later stages which are most impacted by threats in the region [8,9,15]. Misidentifying smaller reproductively-capable adults as non-reproductive subadults in such threat assessments will result in an overestimation of adult life stage survivorship and underestimation of population-level reproductive output, potentially leading to misaligned management priorities [24,49,50].

Using two standard deviations below the mean as an estimate for minimum size at maturity following Stewart et al. [34], our data suggests a revised threshold of 82.8 cm CCL_min_ would be suitable for the Northern Gulf of Mexico Loggerhead Recovery Unit, which is in line with an 82.0 cm threshold based on the Benscoter et al. [65] Gulf Shores data [84]. Such reductions in the established minimum size at maturity are not unique to this recovery unit: Phillips et al. [33] reported a threshold of 83.2 cm CCL_min_ (converted using Turtle Expert Working Group [37]) for loggerhead turtles and 94.8 cm CCL_min_ (converted using Goshe et al. [85]) for green turtles on Florida’s Atlantic coast based on two standard deviations below their almost 40-year mean, as well as declining trends in body size through time. Such an extended time series is not currently available for northwest Florida to investigate whether the observed body size distribution is a recent, persistent, or transient phenomenon. Therefore, sustained capture-mark-recapture efforts are required to support a longer-term trend analysis despite the logistical difficulties associated with monitoring a small population spread over a large geographic area [33,65,72]. If the observed female body size in the region trends smaller through time as in Phillips et al. [33], and if smaller turtles produce fewer eggs as demonstrated here, population-level hatchling production could be negatively affected without a concurrent increase in the number of reproductively active adults [17,31,32,35,44,45,86].

Sustained capture-mark-recapture work will also allow greater exploration of the interactions between female body size, nest site selection, and hatchling production. Maternal behavior can influence hatchling production and physical fitness through nest site and microhabitat selection. Since abiotic conditions drive embryonic development, sex determination, and hatchling fitness, it benefits the nesting female from the perspective of reproductive fitness to identify suitable nesting sites which maximize the production of “fit” hatchlings [51,57,87,88,89,90]. In this study, larger turtles laid larger clutches but also had higher rates of clutch loss due to wave wash-out (Figure 5 and Figure 6). A possible explanation for this is that larger turtles require more energy to crawl further away from the water, resulting in their nests being laid closer to the mean high tide line and thus these nests are more susceptible to the effects of hurricanes or extreme high tides [71,91,92]. This hypothesis runs counter to results from Cabo Verde and Australia where larger loggerhead females nested further from the water, potentially due to increased experience [58,89]. Given logistical limitations, saturation tagging of every individual nesting on St. George Island across the full season was not possible. Therefore, turtles intercepted without pre-existing tags could not be classified as “neophyte” nesters with confidence, regardless of body size. This, combined with the limited sampling window, means the cause of increased wash-out rates for larger turtles on St. George Island is ripe for future investigation. Future studies should consider methods to better quantify turtle experience (e.g., saturation tagging) and expand the sampling period to evaluate possible intra-season differences in the timing of nest deposition by body size and associated degree of overlap with the local hurricane season.

Previous work has highlighted repeatability in individual nest site selection (e.g., [55,57,88]) and the impacts of site selection on hatchling production (e.g., [56,58,89]), but the maternal size distribution is absent from these analyses. Emergence patterns between turtles who are above or below the 87 cm CCL_min_ threshold suggest there may be patterning in the use of the available nesting area on St. George Island (Figure 3 and Figure 4). The center of the island, with a greater prevalence of smaller nesters, tends to have higher rate of artificial lighting, nighttime beach activity, and rates of hatchling disorientation and thus may represent less suitable habitat [67,93]. Remigrant females have been credited with improved selection of suitable nesting habitat through increased experience [58,86]. However, without an obvious mechanism for adult females to evaluate hatchling output as a result of their nest site selection, the role of “experience” may be difficult to quantify. Further studies are needed to investigate spatial trends on St. George Island and any underlying mechanism such as nesting female experience, genetic effects, or competitive exclusion of the smaller size class from higher quality nesting sites or nearshore resting areas, along with any consequences this may have on hatchling production and survival [55,57,86,90,94].

Understanding the distribution of maternal body sizes in a population and any associated relationships this has with nest site selection and threat exposure in space and time may inform future management actions. For example, the trend between nest wash-out and turtle size (Figure 5) is particularly problematic as such erosion results in no hatchling production. Other disturbances including predation and wave overwash have the potential to produce at least a few hatchlings depending on the severity of the disturbance and were not related to female body size in this study [70,71,92,95,96,97]. Because larger turtles on St. George Island lay more eggs and their nests may be more likely to be washed out, management plans should focus on mitigating wash-outs in the area to maximize the population-scale benefits these individuals provide [70]. Such efforts range from the identification and maintenance of suitable nesting habitat to investigations of the logistical and ethical implications of interventionist nest management [71].

Given the observed or potential relationships between female body size, hatchling production, nest site selection, and threat exposure, the exact cause of the broadly observed decline in nesting female size warrants further consideration. For example, reduced habitat suitability or loss due to resource limitations or climate change could restrict juvenile growth rates and encourage early maturation at smaller body sizes [16,22,98,99,100] or direct harvest or incidental capture as bycatch could remove larger individuals from the population [24,101,102,103]. In either of these scenarios, management actions would be required to address the negative causes of the observed decline in body size. However, a positive explanation for reduced body size could be an influx of first-time nesters, which are typically smaller than remigrant nesters, indicating that past and present conservation actions have been effective [18,32,35,52]. At the population level, hatchling production each year is a product of the number of reproductive individuals, the number of eggs laid per individual, and the proportion of eggs lost to various causes. The influx of neophyte nesters may thus compensate for the effect of increased wash-out rate of larger individuals on overall hatchling production, particularly in years with frequent or severe hurricane activity. As this reduction is reported in other recovering rookeries, it may be a common feature of population recovery [18,31,32,34,35,52,104].

## 5. Conclusions

Stage-based population modeling has a long and fruitful history guiding sea turtle conservation (e.g., [8,9,15,64]). Accurately defining stages within models, often based on body size, is critical to the assignment of appropriate vital rates and relative threats [12,20,22,23,24,25,26]. This study supports others in the region [33,65] and globally (e.g., [35]) suggesting that sea turtles are reaching sexual maturity at smaller sizes, with important consequences for mean clutch sizes, nest site selection, and associated hatchling production. Pessimistically, smaller adult sizes may be a consequence of reduced foraging habitat suitability limiting juvenile growth rates [16,22]. Optimistically, the depression in average adult body size may be an artifact of increased recruitment of neophyte nesters within the population [18,35]. Nest counts in the southeastern United States have generally been increasing, suggesting possible recovery, but the exact relationship of nest counts to adult abundance complicates this interpretation [66,105,106]. Additional work confirming population recovery as a significant factor in the observed decline in nesting female body sizes would be welcome news for this threatened charismatic species.

## Data Availability

The data underlying this study may be restricted due to state and/or federal permit requirements. Please direct all data requests to the corresponding author or the Florida Fish and Wildlife Conservation Commission.

## Supplementary Materials

An R Markdown HTML file detailing the analysis process can be downloaded from https://github.com/DrMattWare/NestingSizeOnSGI/ (accessed on 24 November 2025).

## Abbreviations

The following abbreviations are used in this manuscript:

CCL_min_	Minimum curved carapace length
FSU MTRECG	Florida State University Marine Turtle Research, Ecology, and Conservation Group

## Appendix A

**Table A1:** Number of nighttime surveys within each monitoring zone by year.

Zone	2016	2017	2018	2019	2021	2022	2024	Total
A	0	0	0	0	15	13	0	28
B	14	10	14	14	15	13	7	87
C	14	10	14	14	0	0	7	59
D	14	10	14	14	15	13	7	87
F	14	10	14	14	15	13	7	87
G	14	10	14	14	15	13	7	87
H	14	10	14	14	15	13	7	87
I	14	10	14	14	15	13	7	87
J	14	10	14	14	15	13	0	80
